# Quantitative Analysis of Six Phenolic Acids in *Artemisia capillaris* (Yinchen) by HPLC-DAD and Their Transformation Pathways in Decoction Preparation Process

**DOI:** 10.1155/2020/8950324

**Published:** 2020-04-23

**Authors:** Fang Tian, Qun-Jia Ruan, Ying Zhang, Hui Cao, Zhi-Guo Ma, Gai-Lian Zhou, Meng-Hua Wu

**Affiliations:** ^1^College of Pharmacy, Jinan University, Guangzhou, China; ^2^Research Center for TCM of Lingnan (Southern China), Jinan University, Guangzhou, China; ^3^Guangxi University of Chinese Medicine, Nanning, China; ^4^Sun Yat-Sen University, Guangzhou, China; ^5^Kangmei Pharmaceutical Co., Ltd., Shenzhen, China

## Abstract

We aimed to establish a quantitative analysis method of six constituents (5-caffeoylquinic acid, 3-caffeoylquinic acid, 4-caffeoylquinic acid, 1,3-dicaffeoylquinic acid, 3,4-dicaffeoylquinic acid, and 4,5-dicaffeoylquinic acid) in *Artemisia capillaris* (Yinchen) and its decoction by using HPLC coupled with DAD. Besides, the transformation paths of the six constituents were analyzed in decoction preparation processing. The analytical method was fully validated in terms of linearity, sensitivity, precision, repeatability, and recovery and applied to assess the transformation trend and quantitative analysis of the six constituents in Yinchen decoction. The contents of six constituents varied greatly in Yinchen herb and Yinchen decoction, and there were inextricable internal relationships between them. Presumably 3-caffeoylquinic acid was isomerized to generate 5-caffeoylquinic acid and 4-caffeoylquinic acid. Similarly, 1,3-dicaffeoylquinic acid and 3,4-dicaffeoylquinic acid were produced by isomerization of 4,5-dicaffeoylquinic acid. In conclusion, this study provides a chemical basis for quality control of Yinchen decoction, and the changes of selected markers in decoction could give us some novel perspectives to study the relationship between substances and drug efficacy.

## 1. Introduction

Traditional Chinese medicine is frequently used as constituent medications in clinic, whose decoction is the most commonly used dosage form for Chinese physicians, and it is also the oldest and most widely used preparation of Chinese medicinal materials. However, there are few detailed studies on it, especially on the decoction of a single herb, and its connotation and extension are not clear [1, 2]. Yinchen (YC), which belongs to the genus *Artemisia* of the family Asteraceae, is derived from two species, *Artemisia scoparza* Waldst. et Kit. and *Artemisia capillaris* Thunb., and they are defined by two different names according to the different harvest seasons. The aerial part of the plant is harvested in autumn and called “Hua-Yinchen,” and the same part of the plant harvested in spring is called “Mian-Yinchen” [3]. “Mian-Yinchen” is a commonly used decoction pieces in clinical practice.

YC mainly contains phenolic acids, flavonoids, and coumarins [4–7]. And phenolic acids are proved in previous studies to have various biological functions, including antioxidant [8, 9], anti-inflammatory [10], antibacterial [11], cytoprotective [9], hepatoprotective [12, 13], and anti-osteoporosis [14, 15] effects. They are considered as important active ingredients in YC herb. So far, some research studied the analysis of one or several active constituents above in YC herb [16, 17] or herbal formulas containing YC [18–22]. Besides, HPLC-DAD and HPLC-high-resolution MS were applied to study eight organic acids and analyze the possible transformation that might occur between them in preparation of YC extract [23].

The standard decoction of medicinal slices is made of a single herb using the standard process which should be guided by the theory of traditional Chinese medicine, based on clinical practice, and referred to the modern extraction method with a standard process [2]. Although those studies have a certain effect on quality control of YC herbs or its preparation, there is no systematical relevant study on the quality control of phenolic acids between YC herb and YC decoction and their transformation pathways. Therefore, it is necessary to establish a comprehensive analytical method to identify and assay the phenolic acids of YC decoction. And it is investigated whether the transformation of phenolic acids can occur in the preparation of decoction, as well as whether possible transformation pathways exist. It is very crucial to ensure the safety, effectiveness, and quality stability of YC decoction.

The aim of this study was to develop and validate a new analytical method to analyze phenolic acids for quality control of YC decoction using multiple markers, namely 5-caffeoylquinic acid, 3-caffeoylquinic acid, 4-caffeoylquinic acid, 1,3-dicaffeoylquinic acid, 3,4-dicaffeoylquinic acid, and 4,5-dicaffeoylquinic acid. For this purpose, a hyphened method of HPLC coupled with diode array detection was established to analyze the chemical constituents of YC decoction and herbs. In addition, under different heating temperature and heating time, the changes in concentration of the six phenolic acids were studied in water decoction process. The proposed method could be used for qualitative and quantitative analysis and developed as a new tool for the quality evaluation of YC decoction and YC herbs. The concentration changes of the phenolic acids during the decoction process could provide some novel ideas to study the link between substances and drug efficacy.

## 2. Experimental

### 2.1. Chemicals, Reagents, and Materials

**Figure 1:**
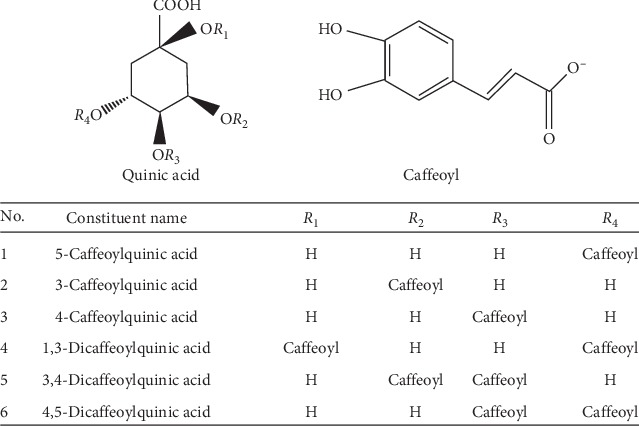
Structures of the six phenolic acid chemical constituents.

The chemical standards of 5-caffeoylquinic acid (batch no. X-014-180410), 3-caffeoylquinic acid (batch no. PRF7041803), 4-caffeoylquinic acid (batch no. Y-067-180425), 1,3-dicaffeoylquinic acid (batch no. E-007-190118), 3,4-dicaffeoylquinic acid (batch no. Y-069-170516), and 4,5-dicaffeoylquinic acid (batch no. Y-070-171216) were purchased from Bio-purify Phytochemicals Ltd.(Chengdu, China). Their structures are shown in Figure 1.

Acetonitrile and formic acid were of HPLC grade which were purchased from Aladdin Chemistry Inc (Shanghai, China). Other reagent solutions were of analytical grade (Shanghai Chemicals, Shanghai, China). Purified water used for a chromatographic mobile phase was purchased from China Resources Yibao Beverage (China) Co., Ltd. (Guangzhou, China).

### 2.2. Plant Material and Sample Pretreatment

The YC herbs were collected from different areas of China. The sources of YC herbs are shown in Table 1. Their botanical origins were identified by Dr. Zhiguo Ma, and they were derived from the dried aerial part of *Artemisia capillaris* Thunb. of the family Asteraceae. Voucher specimens were deposited at the College of Pharmacy, Jinan University. After collection, the samples were dried at 50°C [24].

### 2.3. Preparation of Standard Solutions

A mixed standard stock solution containing 5-caffeoylquinic acid (1), 3-caffeoylquinic acid (2), 4-caffeoylquinic acid (3), 1,3-dicaffeoylquinic acid (4), 3,4-dicaffeoylquinic acid (5), and 4,5-dicaffeoylquinic acid (6) was prepared in 20% methanol. All standard solutions were filtered through a 0.45-*μ*m nylon membrane before injection analysis.

### 2.4. Preparation of Sample Solutions

Preparation of YC decoctions: YC herbs (50 g) were soaked in 600 mL distilled water (1 : 12, w/v) for 30 min and then refluxed by boiling for 1 h. The extract was filtered through a 200-mesh filter cloth. Then, 500 mL of water was added to the filter residue and heated to reflux for 20 minutes. The two filtrates were then combined and concentrated in vacuum to 250 mL, and mixed to obtain a concentration of 0.2 g crude drug/mL. Then, the YC decoction (1 mL) were measured accurately into a 10 mL volumetric flask with stopper, and 10% methanol was added to dilute to the mark, and filtered through 0.45 *μ*m membranes for HPLC injection.

Preparation of YC herb solutions: The dried powders of sieved (24 mesh) YC herb samples were weighed accurately for 1 g into a 100-mL conical flask with stopper, and 50 mL 50% methanol was added to be sonic for 30 min. The conical flask was weighed before and after extraction. The extract was filtered, and 5 mL was accurately measured and placed in a 25-mL conical flask with stopper, and 50% methanol was added, shaken well, filtered, and the filtrate was passed through a 0.45-*μ*m syringe filter (NYLON, Jinlong, Tianjin, China) before injection into the HPLC system for analysis.

### 2.5. Apparatus and HPLC Chromatographic Conditions

Quantitative analysis was conducted using a Thermo UltiMate 3000 HPLC system (USA), equipped with a DAD (190–400 nm), a column temperature controller, and an autosampler. An Agilent Eclipse XDB-C_18_ column (250 mm × 4.6 mm, 5 *μ*m) was used and maintained at 30°C. The mobile phase was 0.1% formic acid aqueous solution (A) and acetonitrile (B) with a gradient program as follows: 0∼20 min, linear gradient 7%–15% B; 20∼30 min, linear gradient 15%–20% B; 30∼35 min, isocratic elution 20% B; 35∼45 min, linear gradient 20%–25% B, and the postrun (5 min) at a flow rate of 1.0 mL/min. The injection volume was 5 μL. Signal monitoring was performed at 330 nm.

### 2.6. Validation of the HPLC Method

#### 2.6.1. Calibration Curves, Limits of Detection, and Quantiﬁcation

To evaluate the linearity of this method, the calibration curve was established from the chromatographic peak area (*Y*) relative to concentration (*X*) of each constituent. The regression equations of six constituents were calculated in the form of *Y* = a*X* + b. The LOD and LOQ for six constituents were estimated at *S*/*N* of 3 and 10, respectively, by injecting a series of dilute solutions with known concentrations.

#### 2.6.2. Precision and Stability

The intraday precision for each constituent was performed in six replicates during a single day. And to confirm the stability, sample 4 was analyzed at 0, 2, 4, 8, 12, 16, 20, and 24 h, respectively. Variability was expressed by RSD (%).

Repeatability was assessed by analyzing the six different working solutions prepared from the same sample (No. 4). Variations of the peak area were expressed as percentage RSD values.

#### 2.6.3. Recovery

The recoveries of the six constituents were tested by adding the mixed standard solutions (100% of the known amount in sample) to YC decoctions, with known amounts. Recoveries were calculated by comparing the determined amount of those standards with the amount originally added.

### 2.7. Transformation Pathways of Six Phenolic Acids at Different Temperatures

The same batch of YC decoction extract was divided into four equal parts and placed in four round-bottom flasks. Electric heating jackets, which were, respectively, set at 40, 60, 80, and 100°C, were heated for 0.5, 1, 1.5, 2, 2.5, and 3.0 h, respectively. The appropriate amount of solution was taken in a different heating time, slightly cooler, and then passed through a 0.45-μm microporous membrane.

## 3. Results and Discussion

### 3.1. Optimization of the HPLC Conditions

**Figure 2:**
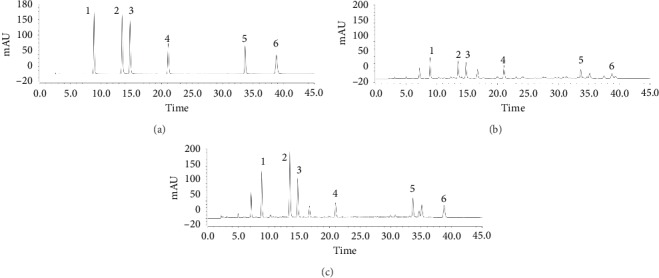
Representative HPLC chromatograms of mixed standards (a), YC herb (b), and YC decoction (c). (1) 5-Caffeoylquinic acid; (2) 3-caffeoylquinic acid; (3) 4-caffeoylquinic acid; (4) 1,3-dicaffeoylquinic acid; (5) 3,4-dicaffeoylquinic acid; (6) 4,5-dicaffeoylquinic acid.

Different mobile phases and columns were investigated to optimize the HPLC analysis. On the basis of several trials, it was found that good resolution and symmetric peak shape were obtained when acetonitrile–0.1%formic acid was selected as a mobile phase. An Agilent TC-C_18_ column (5 mm, 4.6 × 250 mm) and an Agilent eclipse XDB-C_18_ column (5 mm, 4.6 × 250 mm) were tested. The analytical results showed that the latter one was more suitable, exhibiting better peak separation. The detection wavelength was selected at 330 nm according to the analytical results of 3D plots. Representative chromatograms for the standard analytes and the samples are shown in Figure 2.

### 3.2. Identification of Constituents in the Samples by HPLC

According to the reference literatures [23, 25], under the present chromatographic and Chinese medicine standards conditions, six peaks detected from these samples were identified as 5-caffeoylquinic acid (peak 1), 3-caffeoylquinic acid (peak 2), 4-caffeoylquinic acid (peak 3), 1,3-dicaffeoylquinic acid (peak 4), 3,4-dicaffeoylquinic acid (peak 5), and 4,5-dicaffeoylquinic acid (peak 6) by comparison of their HPLC retention times and the ultraviolet spectrum with those of reference constituents.

### 3.3. HPLC Method Validation

The proposed HPLC-DAD method for quantitative analysis was validated by determining the linearity, LOD, LOQ, intraday precisions, stability, and accuracy. As shown in Table 2, all calibration curves showed good linearity within the test ranges, and the overall LODs and LOQs were in the ranges of 0.66–2.52 *μ*g/mL and 2.21–8.40 *μ*g/mL, respectively. The RSD values of intraday variations, repeatability, and stability of the six analytes were less than 2.63%, and the overall recoveries lay between 98.76% and 105.10% with RSD less than 1.83% (Table 3). In addition, the peak purity was investigated by analyzing the DAD; no indications of impurities could be found. Taken together, the results indicated that the established method was accurate for the determination of six chemical markers in the decoctions.

### 3.4. Quantitative Analysis of Yinchen Herbs and Decoctions

The developed HPLC method was applied to the simultaneous determination of the chemical markers including 5-caffeoylquinic acid (1), 3-caffeoylquinic acid (2), 4-caffeoylquinic acid (3), 1,3-dicaffeoylquinic acid (4), 3,4-dicaffeoylquinic acid (5), and 4,5-dicaffeoylquinic acid (6) in samples of YC herbs and decoctions. The results are shown in Table 4 as the mean values of the two replicate injections.

As shown in Table 4, there were remarkable differences among the contents analyzed in different samples both in YC herbs and its decoctions. Among the 12 batches of YC herbs, the content of 4-caffeoylquinic acid had the largest difference, ranging from 0.024% to 0.104%, but the content difference of 3,4-dicaffeoylquinic acid was the smallest, ranging from 0.053% to 0.116%. These differences in chemical constituents might be attributable to multiple factors, such as differences in climate, growing region, and harvest conditions. Among the YC decoctions, the contents of 5-caffeoylquinic acid varied the most, ranging from 0.019% to 0.102%. The smallest content variation was 1,3-dicaffeoylquinic acid, which ranged from 0.012% to 0.029%. The main reason for the differences might be related to differences in the content of the YC herbs that were used to prepare the YC decoction and the preparation process of YC decoction. In addition, the transfer rate of each constituent varied greatly among different sample batches. The average transfer rates of 5-caffeoylquinic acid and 4-caffeoylquinic acid both exceeded 100% and were around 130%, but the average transfer rates of 3-caffeoylquinic acid and 4,5-dicaffeoylquinic acid were only about 15%. Such a large difference that occurred in YC herbs and YC decoctions was probably due to the transformation of the constituents during the water decoction.

### 3.5. The Changes of Six Chemical Constituents in Decoction Process

**Figure 3:**
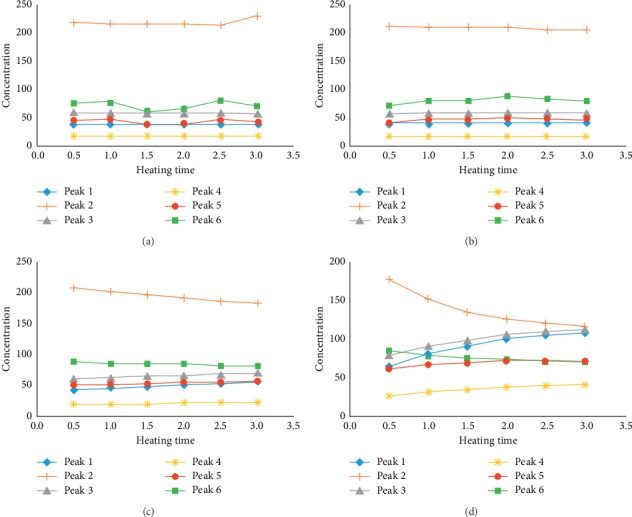
The concentration (*μ*g/mL) change curves of six chemical constituents under different heating temperature and heating time in water decoction process. (1) 5-Caffeoylquinic acid; (2) 3-caffeoylquinic acid; (3) 4-caffeoylquinic acid; (4) 1,3-dicaffeoylquinic acid; (5) 3,4-dicaffeoylquinic acid; (6) 4,5-dicaffeoylquinic acid. (a) 40°C, (b) 60°C, (c) 80°C, and (d) 100°C.

**Figure 4:**
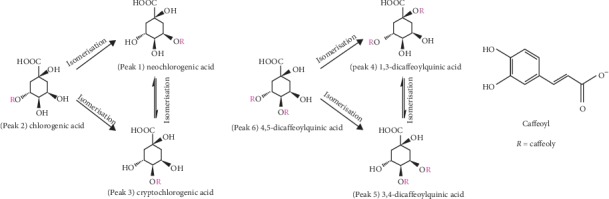
Possible transformation pathways of mono-caffeoylquinic acid (5-caffeoylquinic acid, 3-caffeoylquinic acid, and 4-caffeoylquinic acid) and bis-caffeoylquinic acid (1,3-dicaffeoylquinic acid, 3,4-dicaffeoylquinic acid, and 4,5-dicaffeoylquinic acid).

In order to verify whether there was constituent transformation in the preparation of YC decoction, the prepared YC decoction extract was boiled and refluxed at different heating time and heating temperature. Then, the samples were injected into the HPLC system for analysis. The results are shown in Figure 3. With the extension of the heating time in water decoction process, the concentrations of the six constituents hardly changed at the temperatures of 40°C and 60°C but showed a slight change trend at 80°C. When the temperature was 100°C, there were significant changes in the six constituents, and with the increase of heating time, the increasing trends of the six constituents varied greatly. Among them, the concentrations of 3-caffeoylquinic acid and 4,5-dicaffeoylquinic acid significantly decreased, while the concentrations of 5-caffeoylquinic acid, 4-caffeoylquinic acid, 1,3-dicaffeoylquinic acid, and 3,4-dicaffeoylquinic acid all exhibited an upward trend, and the concentrations of 5-caffeoylquinic acid and 4-caffeoylquinic acid increased significantly. In addition, under heating for 3h, the variation differences among the six constituents were the smallest and tended to be stable. According to the binding sites and numbers of caffeoyl (Figure 1), 5-caffeoylquinic acid, 3-caffeoylquinic acid, and 4-caffeoylquinic acid were mono-caffeoylquinic acids, and they were isomers of each other. Bis-caffeoylquinic acids mainly included 1,3-dicaffeoylquinic acid, 3,4-dicaffeoylquinic acid, and 4,5-dicaffeoylquinic acid, which were also isomers. During the preparation of YC decoction, under the influence of external conditions such as heating temperature and time, the rupture of the caffeoyl would occur, and the substitution position of whom on the quinic acid would be rearranged to produce the corresponding isomer. From the results above, combined with the structures of the six phenolic acid chemical constituents (Figure 1), it could be speculated that 3-caffeoylquinic acid was isomerized to generate 5-caffeoylquinic acid and 4-caffeoylquinic acid, which were also isomerized with each other; similarly, 4,5-dicaffeoylquinic acid produced 1,3-dicaffeoylquinic acid and 3,4-dicaffeoylquinic acid could also be isomerized with each other. The possible transformation paths are shown in Figure 4.

## 4. Conclusion

In this study, the HPLC-DAD method was developed and verified for its qualitative and quantitative analysis of six phenolic acids in YC decoction and YC herbs, namely, 5-caffeoylquinic acid, 3-caffeoylquinic acid, 4-caffeoylquinic acid, 1,3-dicaffeoylquinic acid, 3,4-dicaffeoylquinic acid, and 4,5-dicaffeoylquinic acid. In addition, this method was used to explore the variation of the six phenolic acid constituents in YC decoction under different heating temperature and heating time. The existing literature [23, 25, 26] mainly studied the changes of phenolic acids at a temperature (100°C) during the decoction process. In order to further investigate the sensitivity of phenolic acids to temperature, four heating temperatures (40°C, 60°C, 80°C, and 100°C) were compared in this study. The results show that phenolic acids hardly changed when the temperature was below 60°C, changed when the temperature was 80°C, but the trend was not obvious, and changed significantly when the temperature was 100°C. It shows that the isomerization phenomenon of phenolic acids becomes more and more obvious with the increase of heating temperature. The results can provide us with novel ideas to study the relationship between substance and drug efficacy. Besides, it provides a solid experimental basis for future study of YC formula granules and quality standard.

## Figures and Tables

**Table 1:** Source information of YC herbs (*Artemisia capillaris* Thunb.).

No.	Batch number	Place of origin	No.	Batch number	Place of origin
YC-1	180810	Shaanxi	YC-7	HX18H01	Shaanxi
YC-2	180421	Shandong	YC-8	180401	Henan
YC-3	180301	Shandong	YC-9	20180601	Shandong
YC-4	181001	Gansu	YC-10	171201	Shaanxi
YC-5	18Z0601	Shandong	YC-11	2017120731	Shanxi
YC-6	180809	Shaanxi	YC-12	1809003	Shandong

**Table 2:** Calibration curves, LOD, and LOQ data (*μ*g/mL) of six phenolic acids.

No.	Analytes	Calibration curves^a^	*R* ^2^	Linear range	LOD	LOQ
1	5-Caffeoylquinic acid	*Y* = 0.1718*X* + 0.0524	1.0000	7.640∼458.4	0.66	2.21
2	3-Caffeoylquinic acid	*Y* = 0.1749*X* + 0.1528	0.9999	8.520∼681.2	0.88	2.95
3	4-Caffeoylquinic acid	*Y* = 0.1518*X* + 0.0197	0.9999	8.800∼528.0	0.98	3.27
4	1,3-Dicaffeoylquinic acid	*Y* = 0.1903*X* + 0.0243	1.0000	4.400∼264.0	0.73	2.42
5	3,4-Dicaffeoylquinic acid	*Y* = 0.1282*X* + 0.0132	0.9999	5.640∼338.4	1.38	4.59
6	4,5-Dicaffeoylquinic acid	*Y* = 0.1197*X* − 0.0317	0.9999	6.480∼388.8	2.52	8.40

^a^ *Y* = A*X* + B, where *Y* is peak area and *X* is concentration of the constituents (*μ*g/mL).

**Table 3:** Precision, repeatability, stability, and recovery of six phenolic acids.

No.	Analytes	Precision (RSD %)^a)^	Repeatability (RSD %, *n* = 6)	Stability^a^ (RSD%, *n* = 6)	Recovery^b^ (%, *n* = 6)	
		Intraday (*n* = 6)			Mean	RSD%
1	5-Caffeoylquinic acid	0.06	0.10	0.57	104.64	0.22
2	3-Caffeoylquinic acid	0.27	0.10	1.31	105.10	0.34
3	4-Caffeoylquinic acid	0.25	0.04	1.14	102.82	1.82
4	1,3-Dicaffeoylquinic acid	0.21	0.09	0.45	98.76	1.39
5	3,4-Dicaffeoylquinic acid	0.46	0.84	1.34	103.45	1.79
6	4,5-Dicaffeoylquinic acid	0.29	0.99	2.62	99.68	1.27

^a^RSD (%) = (SD/mean) × 100. ^b^Recovery (%) = 100 × (amount found – original amount)/amount spiked.

**Table 4:** The contents (%) and transfer rate (%) of six constituents (*n* = 2).

Sample no.	1^c^			2			3			4			5			6		
	YC^a^ (%)	YCD^b^ (%)	Transfer rate^d^ (%)	YC (%)	YCD (%)	Transfer rate (%)	YC (%)	YCD (%)	Transfer rate (%)	YC (%)	YCD (%)	Transfer rate (%)	YC (%)	YCD (%)	Transfer rate (%)	YC (%)	YCD (%)	Transfer rate (%)
1	0.039	0.069	175.4	1.024	0.261	25.4	0.064	0.089	138.2	0.045	0.029	63.0	0.088	0.046	52.7	0.310	0.048	15.4
2	0.025	0.034	140.0	0.651	0.185	28.5	0.040	0.049	121.9	0.036	0.028	78.7	0.076	0.040	52.7	0.188	0.052	27.8
3	0.036	0.102	283.7	1.450	0.242	16.7	0.067	0.112	166.0	0.026	0.021	82.8	0.092	0.047	51.3	0.254	0.042	16.5
4	0.033	0.060	184.2	1.237	0.169	13.7	0.052	0.072	136.9	0.018	0.013	72.9	0.077	0.045	58.0	0.198	0.049	25.0
5	0.060	0.055	92.9	1.413	0.174	12.3	0.104	0.068	65.6	0.017	0.018	109.7	0.108	0.049	45.1	0.494	0.062	12.5
6	0.046	0.031	66.9	1.408	0.155	11.0	0.070	0.043	62.1	0.041	0.012	29.4	0.116	0.030	26.2	0.338	0.038	11.3
7	0.040	0.035	88.7	1.075	0.115	10.7	0.067	0.044	65.4	0.027	0.012	44.6	0.097	0.040	41.5	0.442	0.050	11.3
8	0.40	0.049	124.9	1.135	0.183	16.1	0.059	0.064	109.1	0.019	0.015	77.2	0.081	0.044	54.3	0.299	0.051	17.2
9	0.18	0.019	102.6	0.908	0.085	9.4	0.038	0.026	66.5	0.049	0.016	32.3	0.075	0.021	28.4	0.180	0.029	16.3
10	0.046	0.048	104.7	1.023	0.160	15.7	0.057	0.058	103.0	0.045	0.020	44.1	0.067	0.050	74.2	0.271	0.062	22.9
11	0.027	0.043	161.4	0.786	0.080	10.2	0.045	0.045	98.1	0.017	0.013	75.4	0.073	0.035	47.6	0.315	0.033	10.4
12	0.029	0.025	84.3	0.565	0.090	15.9	0.024	0.105	430.6	0.038	0.018	46.6	0.053	0.016	29.5	0.162	0.018	11.2
Average value	0.036	0.048	134.1	1.056	0.158	15.5	0.057	0.064	130.3	0.032	0.018	63.1	0.084	0.039	46.8	0.288	0.045	16.5

^a^YC, Yinchen herb (*Artemisia capillaris* Thunb.). ^b^YCD, Yinchen decoction. ^c^The analysts are the same as in Figure 2. ^d^Transfer rate (%) = quality of index constituents in YC decoction/quality of index constituents in YC herbs × 100.

## Data Availability

The data used to support this study were obtained from the Jinan University, Guangzhou, China, and are available from the corresponding author upon request.

## Abbreviations

HPLC: High-performance liquid chromatography
DAD: Diode array detector
LOQ: Limit of quantitation
LOD: Limit of detection
RSD: Relative standard deviation
YC: Yincen.
